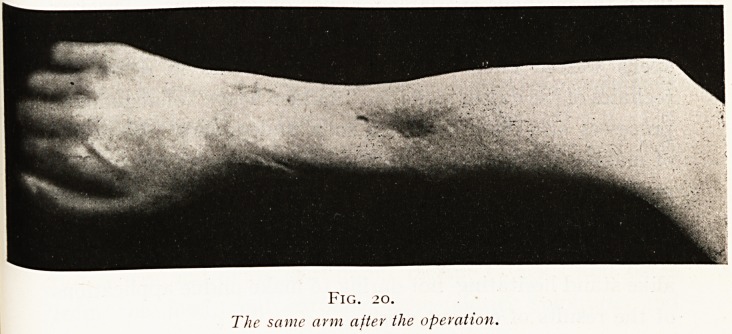# The Long Fox Lecture: The Repair of Bone Injuries

**Published:** 1921-12

**Authors:** Ernest W. Hey Groves

**Affiliations:** Lecturer in Clinical Surgery in the University of Bristol; Surgeon to the Bristol General Hospital


### THE LONG FOX LECTURE:

THE EIGHTEENTH ANNUAL LECTURE ARRANGED BY THE COMMITTEE OF THE LONG FOX LECTURE,

DELIVERED IN THE PHYSIOLOGICAL LECTURE THEATRE OF THE UNIVERSITY OF BRISTOL ON DECEMBER 28TH, 1921.

SIR HENRY GRAY, K.B.E., C.B., *in the Chair.*

BY

ERNEST W. HEY GROVES, M.S., F.R.C.S.,

*Lecturer in Clinical Surgery in the University of Bristol; Surgeon to the Bristol General Hospital.*

ON

### THE REPAIR OF BONE INJURIES.

I FEEL it a very great honour to have been asked to give this Long Fox Memorial Lecture, both because it associates me in the first place with one who was a great clinician and one who by his interest in research has even to this day stimulated interest in medical science, and also because it associates me with an honourable line of predecessors.

Five thousand years ago a young man in Egypt suffered a fracture of his radius, and was treated in the most approved fashion of the time by having his arm splinted by the central rib of a palm leaf. Shortly after he met his death, and his bone, still supported by the primitive splint, is now to be seen in the Royal College of Surgeons. Eight hundred years ago in a village of Wessex a woman who had fractured her humerus had it bandaged up and then surrounded by two plates of thin copper. The remains can still be seen in the Museum at Reading.

This is a fair representation of the treatment of bone injuries up till twenty-five years ago, when two great factors were introduced which greatly changed our conception of the actual treatment of bone repair. One, the introduction of the X-rays ; the other, the general adoption of Industrial Insurance. Both these factors served to show that bone repair was often unsatisfactory, and led to a very great crippling and loss of function.

During the last generation advance has been along four roads. In the first, the treatment of fractures in which there has been little or no displacement has been by massage and early movement rather than splinting, and the improvement produced by this practice, chiefly associated with the name of Lucas-Championniere, has not merely been due to the special type of massage and early movement, but also to the negative factor of the absence of immobilisation. The second road along which knowledge developed is that for gross displacements direct open operative treatment has been devised. Thirdly, that for the great majority of fractures with serious displacement traction systems, by which the ends of the bones are held apart from one another, have been introduced. Lastly, for those cases which either by injury or by the action of disease present a loss of substance the treatment by bone grafting has been devised and brought to a state of practical utility.

It will first of all be useful to discuss one or two of these advances in knowledge from an experimental point of view, and then to follow up the facts into the clinical realm.

The first observation (Fig. 1) concerns the union of a complete fracture by means of a short metal plate attached by screws. You will observe that after a lapse of a few weeks the bones have come apart and are much angulated and deformed This is a fair representation in a somewhat extreme form of what commonly happens when too much reliance is placed upon the fixation of a fracture by a short plate. That is to say, unless it is supplemented by external splinting it cannot keep the bone in position. The second observation (Fig 2) shows that if a large metal peg be placed inside the bone of a size to act as an efficient splint firm union will take place. A further comparison between these two specimens, the one united with a short plate and the other with a large peg, shows that in the first there was sepsis and discharge, whereas in the second healing was perfect. This difference is not due to the position of the large peg inside the bone, as you will see by the consideration of another specimen (Fig. 3) in which a fracture of the femur has been fixed by a plate two-thirds of the length of the bone and by a number of perforating pins. Here too union is absolutely strong and healing has been perfectly smooth.

The lesson from the comparison of such experiments seems to be perfectly clear, and it is that metal substances may be used for the repair of bone and be tolerated by the tissues perfectly, but it is essential that they should be strong enough, large enough, and sufficiently firmly fixed to restore absolute continuity to the mended bone. If the bones are weakly tied together so that movement occurs between them, then the metal substances will become loosened and a septic sinus will be formed.

Another experiment shows that the most perfect artificial support for simple fractures of the long bones consists in a traction appliance, which whilst firmly holding the two extremities of the bone does not directly interfere with the seat of fracture. Bones tied in this way show the most

FIG. 1.
*Cat's femur, 4 weeks after union by short plate and screws.*

FIG. 2.
*Cat's femur, 6 weeks after fracture, fixed by means of a steel peg.*

FIG. 3.
*Cat's femur, 8 weeks after fracture, united by a long plate and perforating pins.*

perfect type of quick and yet complete bone repair. (Fig. 4.) There is neither non-union, callus excess, nor deformity, and there can be no doubt that such a system should be the ideal one for the treatment of human fractures.

If, however, a gap exists in one of the long bones, at any rate in an adult, this gap usually remains permanently ununited. If a portion of the shaft of a bone be removed, even though the periosteum

FIG. 4.

*Cat's tibia, 4 weeks after fracture, united by a double transfixion appliance.*

FIG. 5.

*Cat's tibia, 4 weeks after removal of a piece of the shaft. Bone chips were placed in the gap.*

remain, and if the gap is filled up by means of a number of bone chips taken from the excised portion, at the end of several weeks not only will union be absent but a great number of the bone chips will be absorbed. (Fig. 5.) This fact is one of great importance, and if it were not true then the filling up of defects of living bone would always be most readily accomplished by packing the gap with scraps of bone tissue taken from some redundant bone, such as a rib. There is only one satisfactory way of filling such a gap, and that is by placing in it a piece of living bone of the full size necessary to completely fill the gap; and further, it is not sufficient

Fig. 6.

*Graft (a) fixed into a gap in a cat's tibia by metal pins and washers, with firm union.*

Fig. 7.

*Graft (a) tied into a gap in cat's tibia with catgut, with marked displacement.*

merely to drop the graft into its place, but it must be put into wide contact with the adjacent bone and fixed firmly to it. If this fixation be carried out by means of fitting and shaping, it is the ideal method; but if not, a graft will grow in place much better if it is fixed to its bed by metal pins or screws than if it is merely loosely tied by sutures of catgut. (Figs. 6 and 7.)

Another observation will show that the graft used must be of the full size of the gap it is to fill, and that it cannot be expected that a small graft will grow so as to fill a large place. (Figs. 8 and 9.)

FIG. 8.

*A gap fracture of the radius into which a small piece of tibia has been loosely dropped. No union has occurred.*

FIG. 9.

*Same case as Fig. 8. The gap has been filled by a large graft, which has grown into place.*

There are two vital processes constantly going on in living bone, and these also occur in grafted bone. These are the formation of new bone and also the absorption of old bone. We cannot tell exactly what determines the proportion between these two processes, but it is possible to prove quite definitely that bone growth, that is the formation of new bone, will never take place in conditions of health unless the bone concerned is subjected to stress, that is, actual transmitting of weight or force. But a bone graft not only acts as a living tissue which under suitable conditions will build up new bone, but it also acts as a strut and a scaffold along and around which living bone spreads. This second function of a graft can be undertaken by dead bone as well as by living, and there are certain conditions in which dead bone

FIG. 10.

*Two tibias of a cat. Into the left a living and into the right a dead bone graft has been fixed, with almost identical results.*

is actually better than living for the purposes of a graft. (Fig. 10.)

Very briefly stated, the above are some of the simple facts which may be learned from experimental results, and it will now be possible to see how such general principles are illustrated in the clinical repair of bone injuries.

That a living bone graft has certain definite capacity for growing and for resisting disease is shown by various observations. One of the most striking of these is the condition

FIG. 11.

*Central sequestrum in a graft placed in a gap fracture of the radius.*

in which a living bone graft will often throw off a portion of its substance as a sequestrum, and in one striking instance which I have observed, this dead portion was in the centre of the living graft, so that it must have been separated by the vital action of the bone cells in the graft itself. (Fig. 11.) Another common manifestation of inherent vitality in the graft is presented by the formation of callus union in the repair of a fracture of the graft.

It has often been suggested that the periosteum surrounding the shafts of bone has an inherent power of forming new bone, and that by taking flaps of this membrane bridges may be formed which will cover in gaps and defects in the bone ; but such an idea is founded upon false observations, and any attempt to fill up a gap formed by periosteal flaps will be doomed to failure, because such flaps after throwing out minute pieces of bone may be then absorbed long before continuity has been restored.

Another frequent error which is made is the attempt to fill up gaps in the bone of human beings by means of comparatively small chips taken from other parts. It has already been pointed out that a simple experiment will prove the futility of this, and yet you will see in the series of pictures before you many instances where a small fragment of bone has been loosely thrown into a gap fracture. Some weeks later, non-union still existing, a metal plate has been added (see Figs. 8 and 9) and fixed by screws. Non-union still persists, and usually, as the bones are wobbling about upon one another, the metal plate and its screws have to be removed because of the resulting sinus. Ultimately, such a case will either present permanent non-union, or else vital restitution of continuity can only be brought about by taking a large live piece of bone and fixing it securely to the fragments on either side.

This actual problem of the mechanical fixation of the graft to its bed has occupied much time and attention. Many methods are possible, some being particularly suited for some bones and others for others. For example, in the gap fracture of one of the forearm bones the graft may be shaped something like a cricket bail, the two small ends of which are firmly thrust into the marrow cavity of the fragments at each side of the gap. (Fig. 12.) In bones such as the tibia a deep groove is cut in each fragment, and into this groove a strong piece of bone from the opposite leg is firmly fitted by a mortise inlay. In certain situations, such as the upper end of the femur or the lower end of the humerus, a graft may be thrust right into the centre of the bone by boring a hole right through the articular extremity of the tubular bone, and then using the graft as a stout central

FIG. 12.
*"Cricket bail" graft inserted into a gap in the radius.*

nail which is driven through one end of the bone into the main shaft. (Figs. 13 and 14.)

Again, in a bone like the humerus, which has nothing but soft tissues to support it, the mechanical problem of fixing the graft will present some difficulty. After a period of some months, when a gap has existed in the humerus, the bone will have become so thin by atrophy that nothing is left except a fragile shell. Any cutting into the surface of such a tubular bone will rob it of its only element of strength. Therefore in such a case the graft must be thrust up as a peg firmly fitted into the hollow marrow cavity. In order to give this peg an extra thickness at the site of the gap the grafting is done by two pieces. One is thrust into the upper fragment, the other into the lower, each graft being allowed to project to a distance equal to that of the gap, and here the two projecting pegs are fitted together and fixed into position. (Figs. 15 and 16.)

The reaction of living bone to external force and the need of stress in order to make bones grow is seen very well in the case of bones of children. When the tibia has been destroyed in its central part by acute inflammation, although

FIG. 13.
*Graft used as a nail to fix a fracture of the upper end of the femur.*

FIG. 14.
*Graft used as a nail to fix the lower end of the humerus to the shaft.*

the two extremities of the bone remain and live, and although these extremities contain the growing points of the bone, yet no growth takes place, the reason being that the stimulus of force transmitted from one end of the bone to the other

FIG. 15.

*Gap fracture of humerus.*

FIG. 16.
*Same case shown in last figure, treated by a two-piece graft.*

is absent. If in such a case a long strut of living bone be taken and thrust firmly into the two remaining extremities of the original bone, then within a few months active growth of bone takes place, not only in respect of thickness in the graft but also in respect of the length of the whole bone.

Just as blood pressure is necessary for the circulation and as movement is necessary for the vitality of muscle, so stress and strain are the essential elements necessary for health and growth of bone.

The purely mechanical value of the restoration of continuity is shown in the case of a child from whom the central portion of the humerus had been resected for fibro-cystic disease. This operation involved the removal of 3 inches of the entire thickness of the shaft of the bone, but the vascular periosteum was preserved. At the time of the operation continuity was immediately restored by a two-piece graft made of boiled beef bone, and you will observe that within a few weeks of the operation the new shell of bone is already to be seen in the skiagram, and that within one year the contour of the original bone has been perfectly re-formed. (Fig. 17.)

What now has been gained in such a case by the implanting of the foreign bone into the human tissues, because clearly here all the new bone is human bone produced by human tissues? The gain has been twofold. First, that from within four weeks of the time of the operation the child could use his arm, and therefore practically his disability

Fig. 17.

*Humerus of a boy aged 12, one year after resection of 3 inches of the shaft for cystic disease, with union by a two-piece beef bone graft.*

was at an end. Secondly, by uniting the two ends of the bone firmly that essential active force was provided which alone can stimulate healthy and rapid new bone growth.

It is a somewhat remarkable reflection on the mistaken idea which has sometimes been held regarding the dangerous nature of dead bone, to think that one can deliberately bury a large piece of bone which is not only dead, but which is not even human, in the skeletal tissues, that such tissue will be tolerated, then incorporated, and lastly slowly absorbed. The various stages of such transmutation of beef bone into human bone can be seen in the X-ray pictures of these cases taken at various periods.

Unfortunately, there are sometimes cases where owing to sepsis or to extensive injury of the soft parts a gap in a bone cannot be made good. In some such cases we may, by the exercise of ingenuity, substitute one bone in place of an adjacent bone. Thus, for example, the fibula may be

FIG. 18.

*Forearm of a man who had a gap fracture of the radius.*

substituted for the tibia, that is to say, may be made to take the place which had been occupied by the larger bone.

Another example of bone substitution is afforded by cases of gap fracture of the radius in which grafting has failed for one reason or another. This is seen in the case of a man who had lost a large part of his radius and in whom nothing served to restore its continuity. (Figs. 18, 19, and 20.) The result was that his hand, losing its skeletal support,

FIG. 19.
*Showing the transplantation of the shaft of the ulna into the lower end of the radius.*

FIG. 20.
*The same arm after the operation.*

became so much deflected as to be weak and useless. It seemed hopeless to attempt a further graft, and the patient was unwilling to try. The end of the ulna was therefore removed and the shaft of this bone brought across to the middle of the forearm and thrust into the radius, thus forming a central skeleton to the forearm in place of the paired bone skeleton, and you will see that this gives a symmetrical hand of considerable strength. He has been trained lately as a photographer, and with this hand actually prepared the lantern slides illustrating this lecture.

---

### THE RÔLE OF DILUTE ACIDS IN INFECTION.

BY

I. WALKER HALL AND A. D. FRASER.

## Figures and Tables

**Fig. 1. f1:**
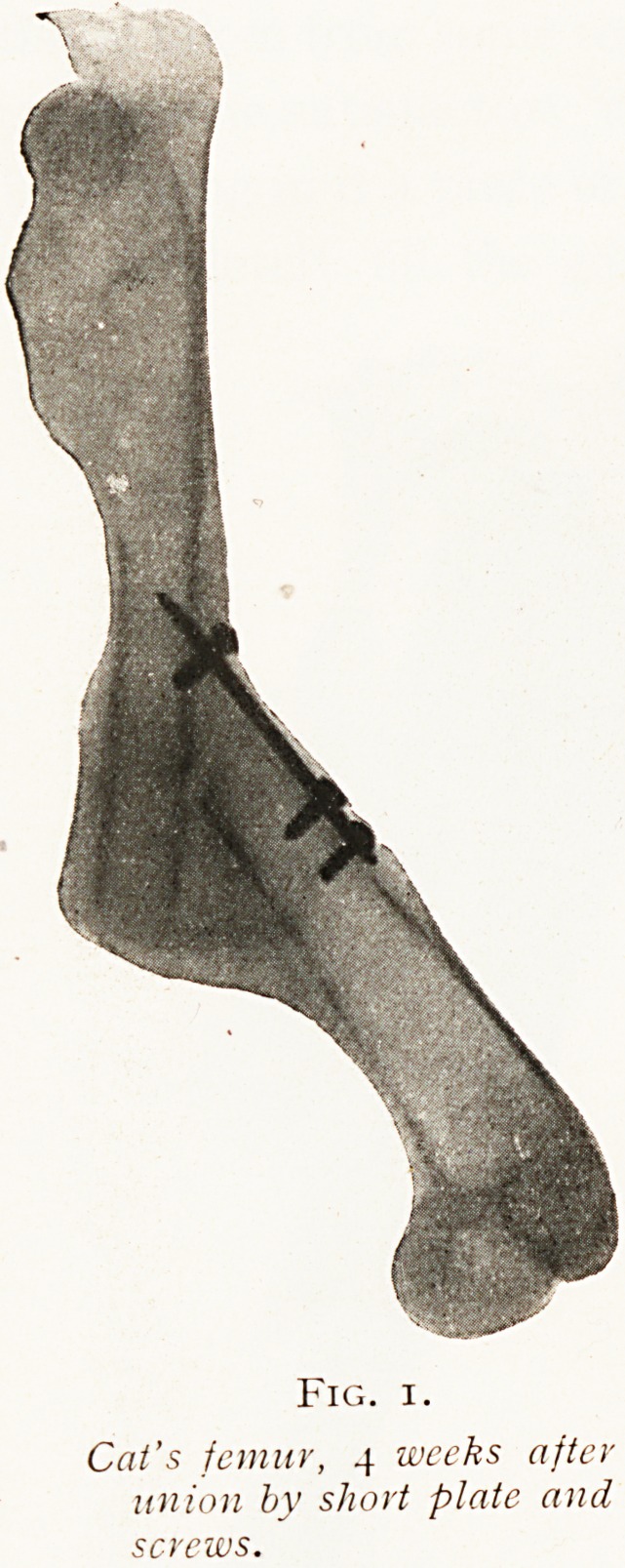


**Fig. 2. f2:**
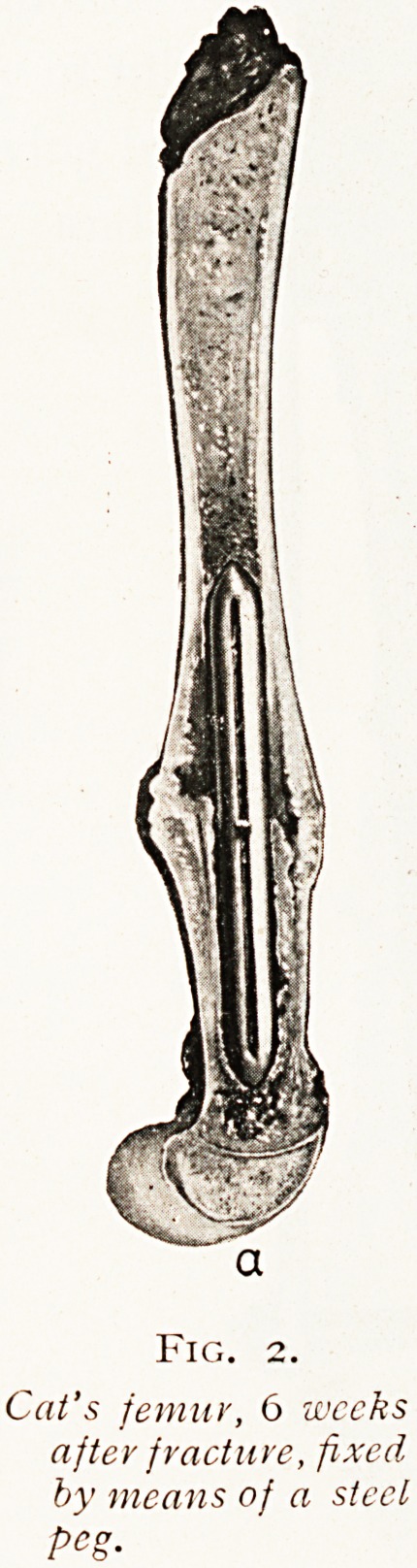


**Fig. 3. f3:**
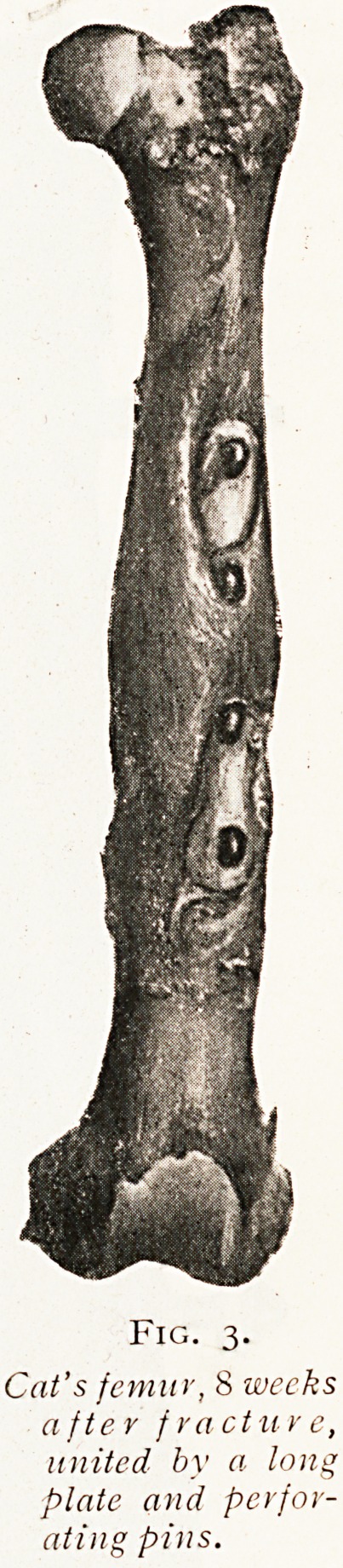


**Fig. 4. f4:**
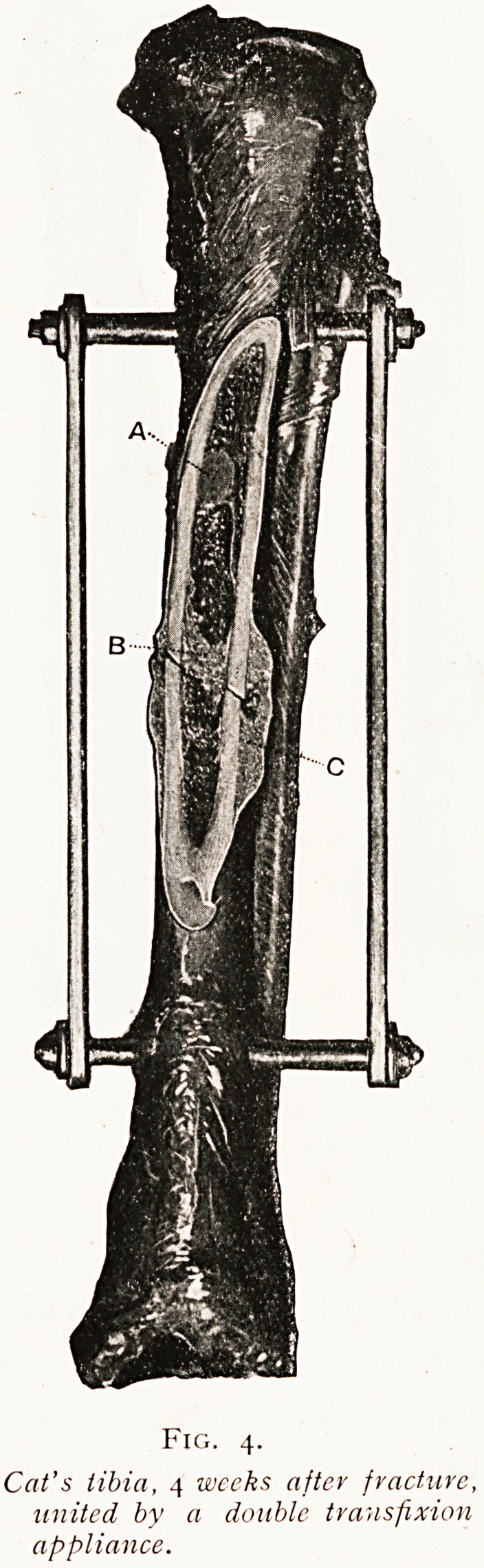


**Fig. 5. f5:**
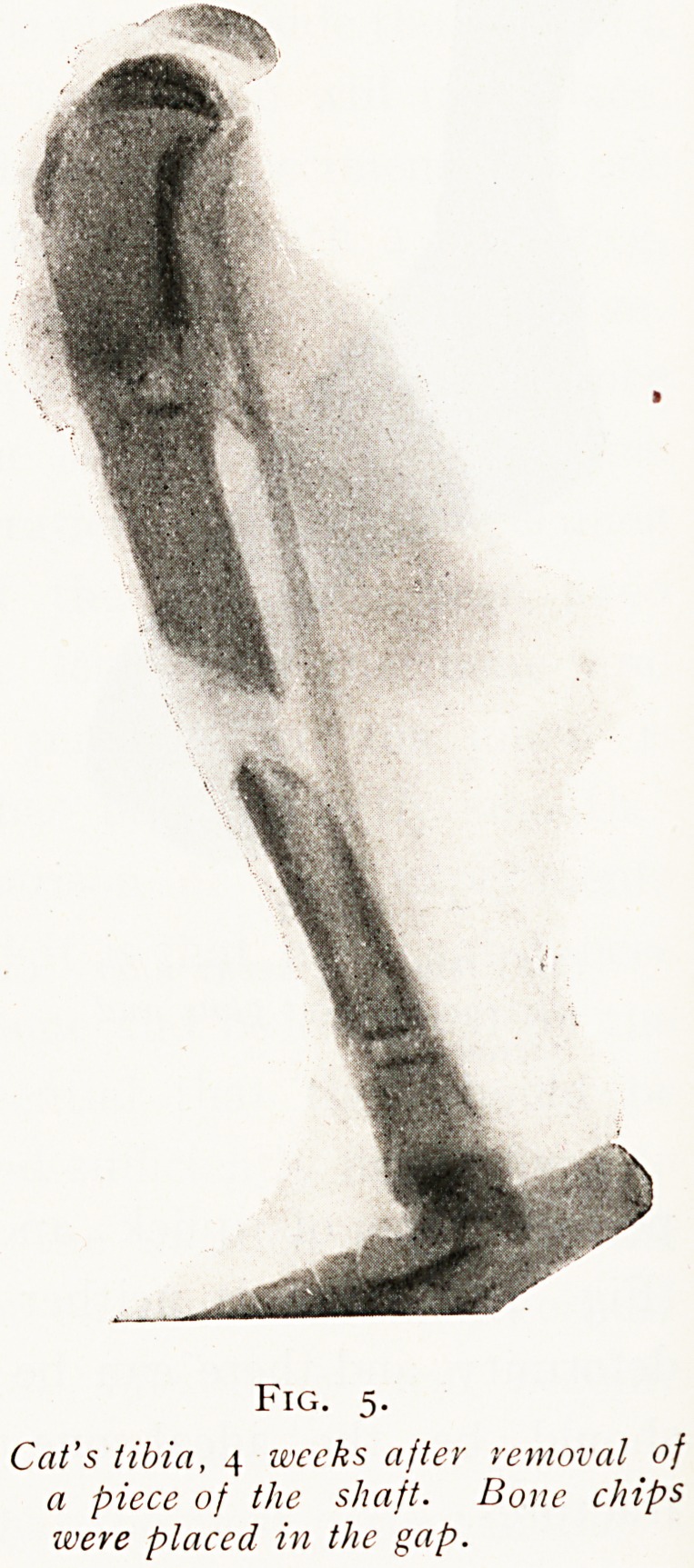


**Fig. 6. f6:**
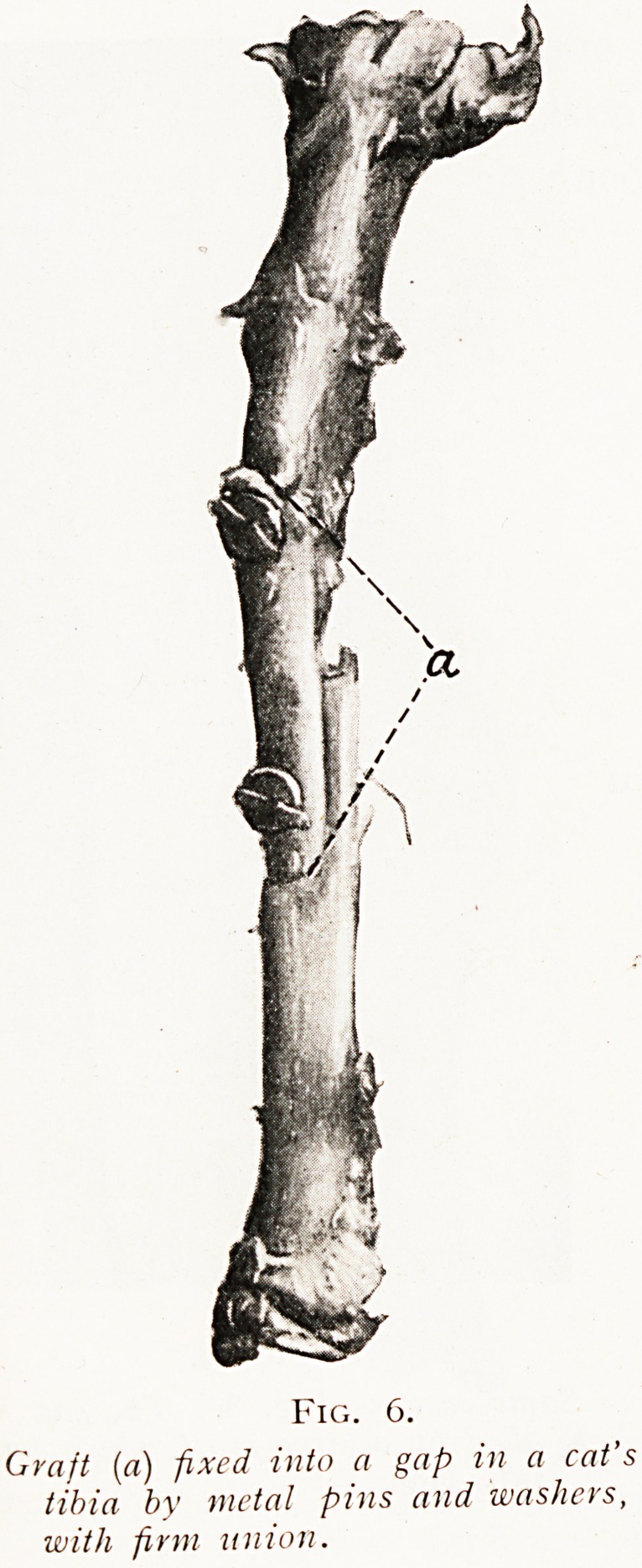


**Fig. 7. f7:**
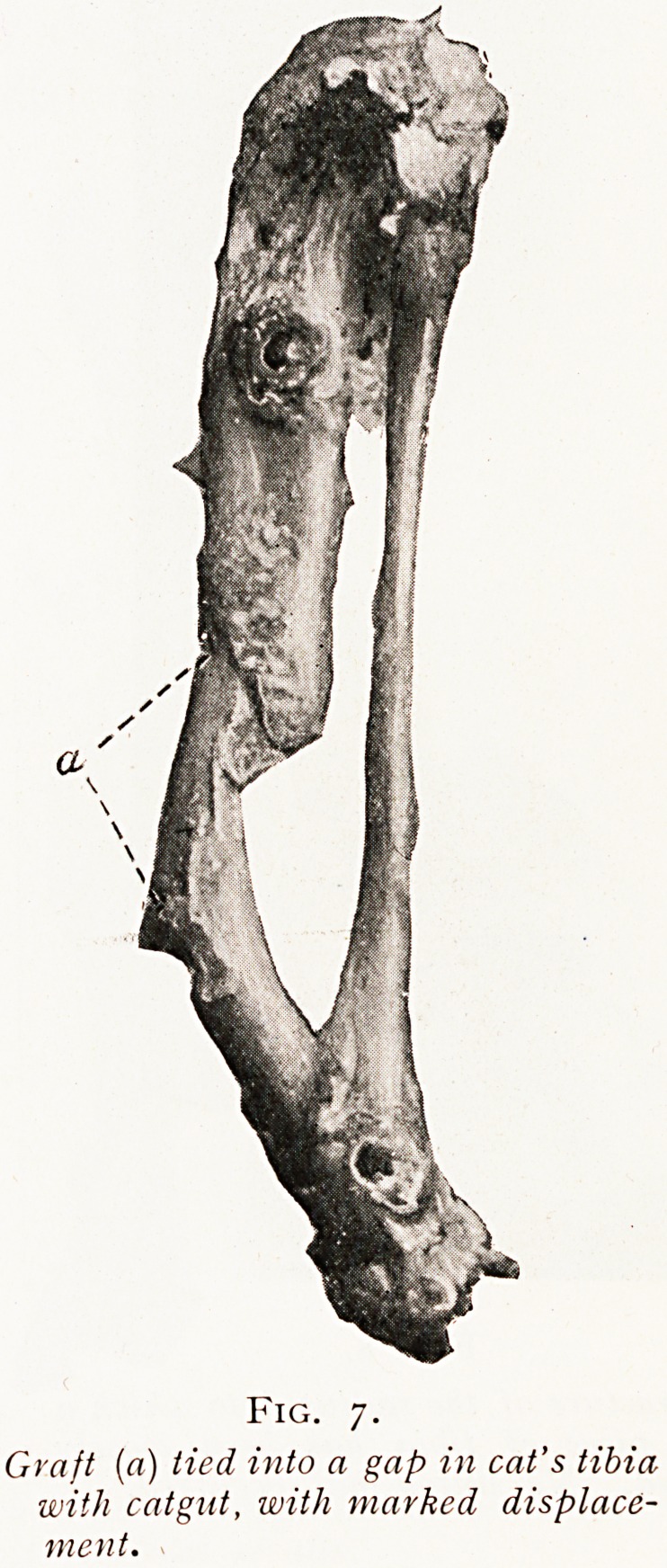


**Fig. 8. f8:**
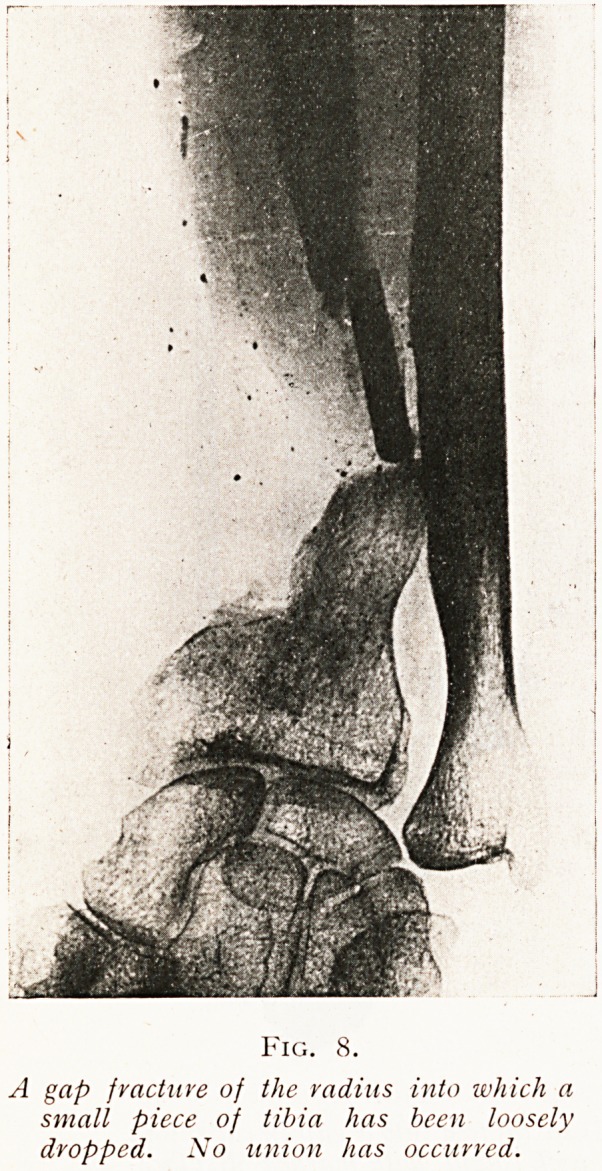


**Fig. 9. f9:**
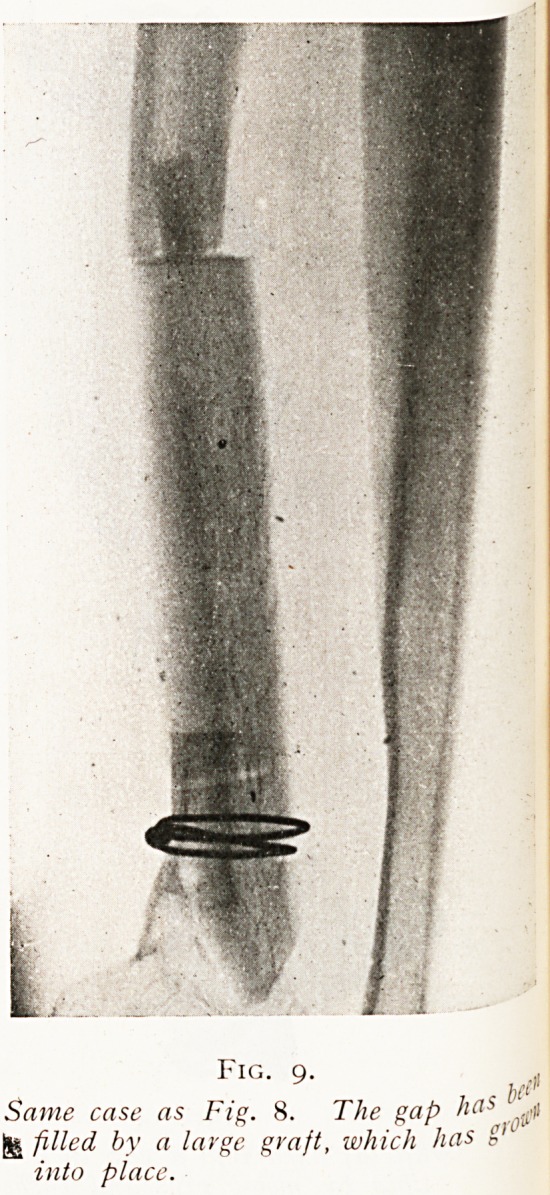


**Fig. 10. f10:**
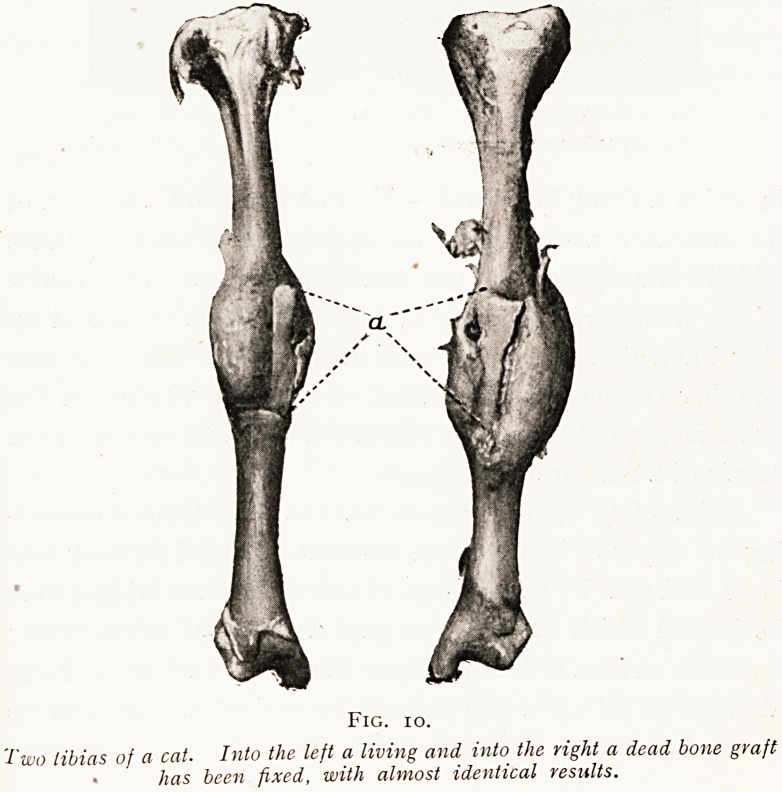


**Fig. 11. f11:**
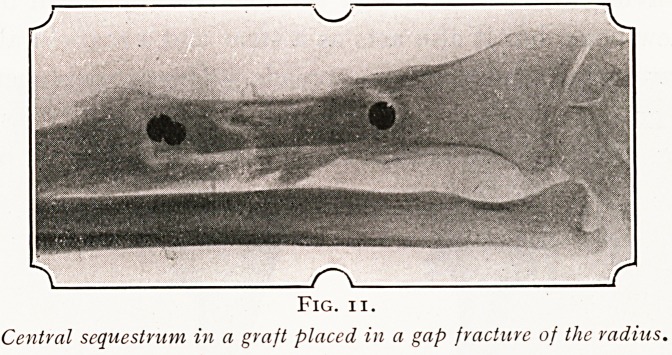


**Fig. 12. f12:**
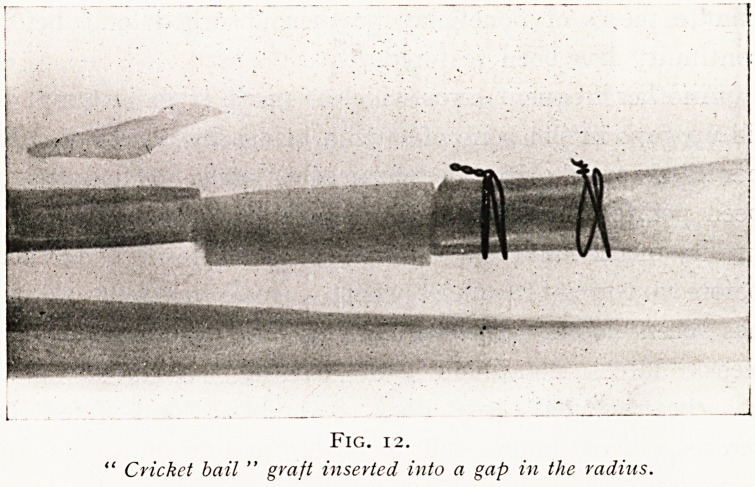


**Fig. 13. f13:**
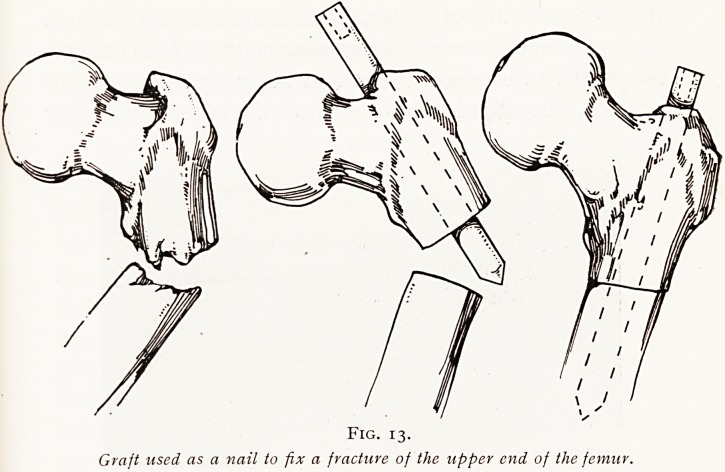


**Fig. 14. f14:**
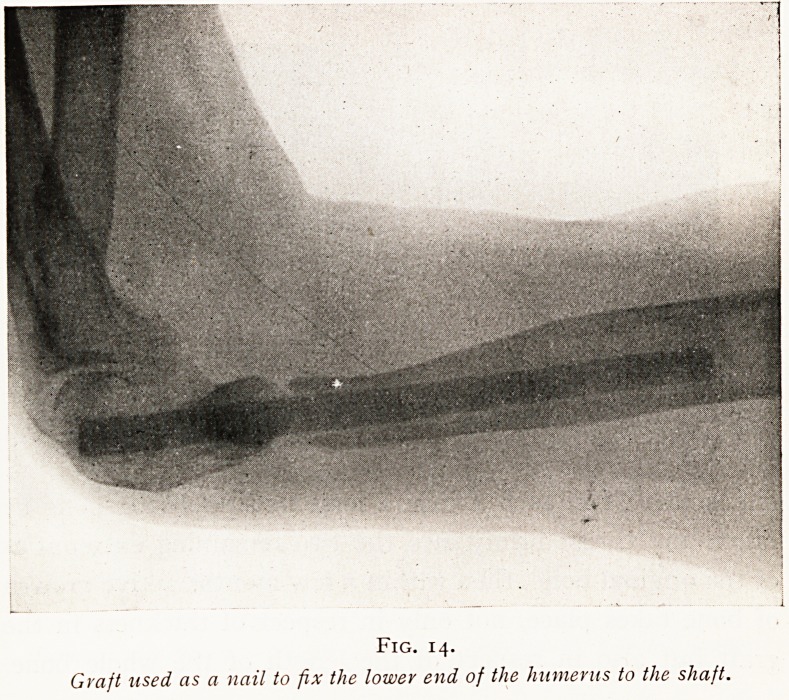


**Fig. 15. f15:**
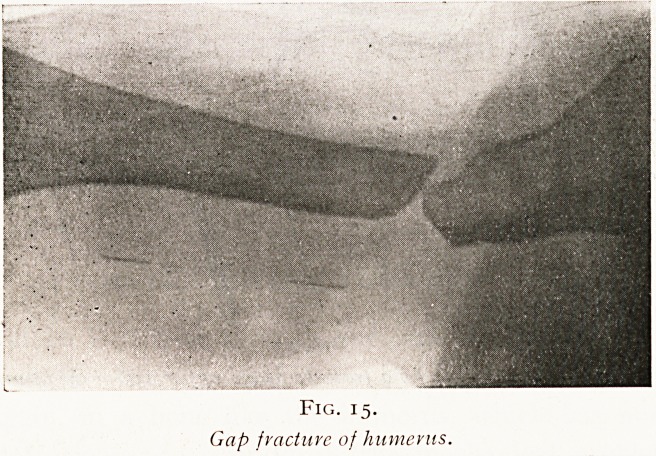


**Fig. 16. f16:**
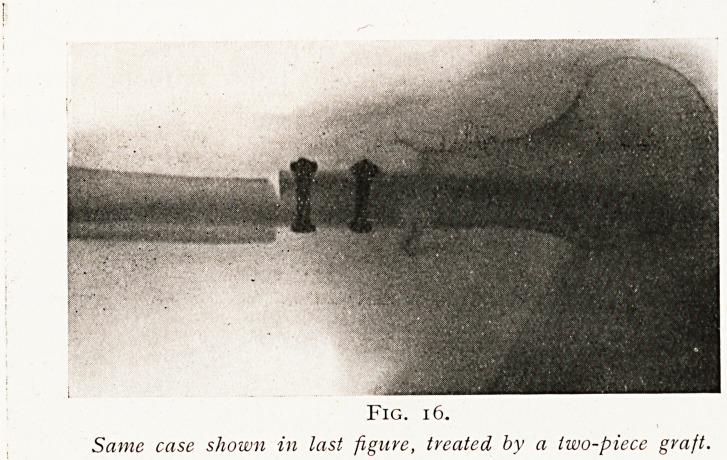


**Fig. 17. f17:**
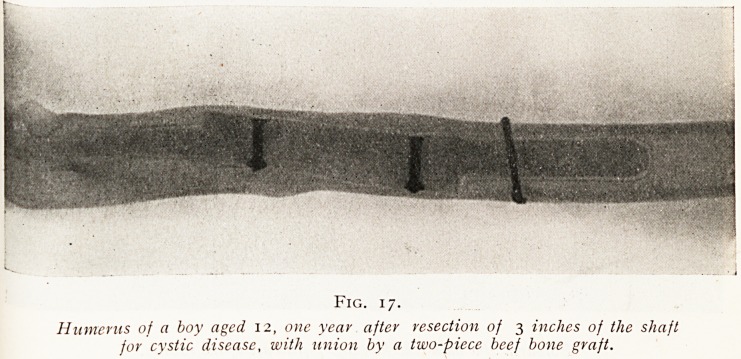


**Fig. 18. f18:**
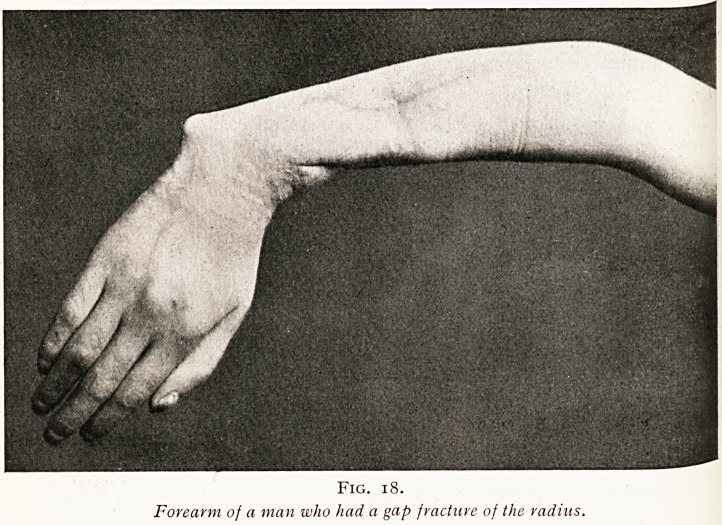


**Fig. 19. f19:**
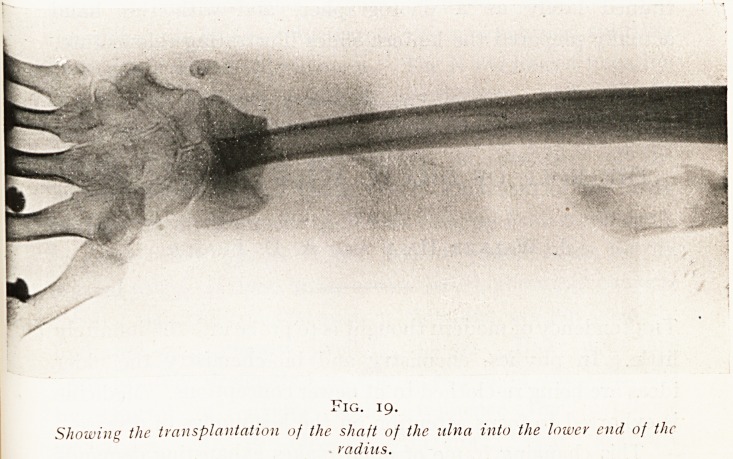


**Fig. 20. f20:**